# Entropy Based Data Expansion Method for Blind Image Quality Assessment

**DOI:** 10.3390/e22010060

**Published:** 2019-12-31

**Authors:** Xiaodi Guan, Lijun He, Mengyue Li, Fan Li

**Affiliations:** 1 School of Information and Communications Engineering, Xi’an Jiaotong University, Xi’an 710049, China; gxd1997@stu.xjtu.edu.cn (X.G.); lijunhe@mail.xjtu.edu.cn (L.H.); lmy1882923@stu.xjtu.edu.cn (M.L.); 2 Guangdong Xi’an Jiaotong University Academy, Foshan 528300, China

**Keywords:** deep neural network, entropy, data expansion, blind image quality assessment, saliency and distortion, human visual system, declining quality

## Abstract

Image quality assessment (IQA) is a fundamental technology for image applications that can help correct low-quality images during the capture process. The ability to expand distorted images and create human visual system (HVS)-aware labels for training is the key to performing IQA tasks using deep neural networks (DNNs), and image quality is highly sensitive to changes in entropy. Therefore, a new data expansion method based on entropy and guided by saliency and distortion is proposed in this paper. We introduce saliency into a large-scale expansion strategy for the first time. We regionally add distortion to a set of original images to obtain a distorted image database and label the distorted images using entropy. The careful design of the distorted images and the entropy-based labels fully reflects the influences of both saliency and distortion on quality. The expanded database plays an important role in the application of a DNN for IQA. Experimental results on IQA databases demonstrate the effectiveness of the expansion method, and the network’s prediction effect on the IQA databases is found to be improved compared with its predecessor algorithm. Therefore, we conclude that a data expansion approach that fully reflects HVS-aware quality factors is beneficial for IQA. This study presents a novel method for incorporating saliency into IQA, namely, representing it as regional distortion.

## 1. Introduction

With the current state of development of multimedia technology, a large number of videos and images are being generated and processed every day, which are often subject to quality degradation. As a fundamental technology for various image applications, image quality assessment (IQA) has always been an important issue. The aim of IQA is to automatically estimate image quality to assist in the handling of low-quality images during the capture process. IQA methods can be divided into three major classes, namely, full-reference IQA (FR-IQA) [1,2], reduced-reference IQA (RR-IQA) [3], and no-reference IQA (NR-IQA), based on whether reference images are available. In most cases, no reference version of a distorted image is available; consequently, it is both more realistic and increasingly important to develop an NR-IQA model that can be widely applied [4]. NR-IQA models are also called blind IQA (BIQA) models. Notably, deep neural networks (DNNs) have performed well in many computer vision tasks [5,6,7,8], which encouraged researchers to use the formidable feature representation power of DNNs to perform end-to-end optimized BIQA, an approach called DNN-based BIQA. These methods use some prior knowledge from the IQA domain, such as the relationship among entropy, distortion and image quality, to attempt to solve IQA tasks using the powerful learning ability of neural networks. Accordingly, there is a strong need for DNN-based BIQA models in various cases where image quality is crucial.

However, attempts to use DNNs for the BIQA task were limited due to the conflicting characteristics of DNNs and IQA [9]. DNNs require massive amounts of training data to comprehensively learn the relationships between image data and score labels; however, classical IQA databases are much smaller than the computer vision datasets available for deep learning. An IQA database is composed of a series of distorted images and corresponding subjective score labels. Because obtaining a large number of reliable human-subjective labels is a time-consuming process, the construction of IQA databases requires many volunteers and complex, long-term experiments. Therefore, expanding the available number of distorted image samples and labels that fully reflect human visual system (HVS)-aware quality factors for training is a key problem for DNN-based BIQA.

Based on the baseline datasets considered for expansion, the current DNN-based BIQA methods can be divided into two general approaches. The first approach is to use the images in an existing IQA dataset as the parent samples; we call this approach small-scale expansion. In this case, the goal of expansion is achieved by dividing the distorted images from the IQA dataset into small patches and assigning to each patch a separate quality label that conforms to human visual perception. The second strategy is to expand the number of distorted images by using another, non-IQA dataset as the parent dataset; we call this approach large-scale expansion. In this approach, nondistorted images from outside the IQA dataset are first selected; then, distortion is added to these images based on the types of distortion present in the IQA dataset to construct new distorted images on a large scale. Then, the newly generated distorted images are simply labeled with different values that reflect their ranking in terms of human visual perception quality to achieve the goal of expansion.

The small-scale expansion strategy relies on division. The initial algorithm [10] assigns the score labels of the parent images to the corresponding small patches and then uses a shallow CNN to perform end-to-end optimization. The small patches and their labels are input directly to the network during training, and the predicted scores for all the small patches are averaged to obtain the overall image score during prediction. However, this type of expansion is not strictly consistent with the principles of the HVS. Previous studies have shown that saliency exerts a crucial influence on human-perceived quality; thus, saliency should be considered in IQA together with distortion and content [11,12,13]. These studies have shown that the human eye tends to focus on certain regions when assessing an image’s visual quality and that different regions have different influences on the perceived quality of a distorted image. Therefore, it is not appropriate for all patches from a single image to be assigned identical quality labels because local perceptual quality is not always consistent with global perceptual quality [14,15]: the uneven spatial distortion distribution will result in varying local scores for different image patches. Thus, many works have attempted to consider this aspect of the problem. The saliency factor was first considered in DNN-based BIQA algorithms. The authors of [16,17] still assigned identical initial quality labels to the small patches, but the predicted scores for all small patches were eventually multiplied by different weights based on their saliency to obtain the overall image scores, thereby weakening the influence of patches with inaccurate labels in nonsalient regions on the overall image quality. In [18,19], strategies based on proxy quality scores [18] and an objective error map [19] were used to further improve the accuracy of the labels for different patches. All these strategies further increased the accuracy of this type of expansion and led to better predictions, confirming that the joint consideration of the influence of saliency and distortion on image quality more comprehensively reflects HVS-related perceptual factors. However, division strategies have obvious inherent drawbacks. First, because expansion is applied only to the existing distorted images in the IQA database (the expansion parent), the diversity of the training sample contents is not increased. The different levels of quality influenced by saliency and distortion must already be present in the training dataset, but it is difficult to claim that a typical small IQA database can comprehensively represent the influence of HVS factors on quality; hence, such methods are easily susceptible to overfitting. Second, there is a tradeoff between the extent of expansion achieved and the patch size. When the patch size is too small, each individual patch will no longer contain sufficient distorted semantic information for IQA, thus inevitably destroying the correlations between image patches. In contrast, a large patch size results in smaller-scale expansion, meaning that only a shallow network can be used for training. Moreover, the generated saliency-based patch weights will show large deviations from the real salient regions.

To avoid dividing the images in the IQA database while still not requiring human labeling, the large-scale expansion strategy instead involves creating new distorted images by adding distortion to a large number of high-definition images obtained from outside the IQA database. Separate values that reflect the overall quality level are assigned to each distorted image obtained from each original parent image. Because the labels of the newly generated images are not direct quality scores, the expanded database is used only to pretrain the DNN, which is then fine-tuned on the IQA database. This approach alleviates the training pressure placed on the small IQA dataset and successfully avoids the drawbacks of division encountered in the small-scale strategy because the number of labeled training images is expanded by a large amount, increasing the diversity of the training sample content. Such unrestricted, large-scale expansion also makes it possible to use deeper networks; in fact, a deep model pretrained on an image recognition task could also be used to further enhance the effect. This large-scale expansion approach was developed over the past two years, and it showed a much better effect than small-scale expansion algorithms. However, large-scale expansion also has some significant shortcomings. Although the newly added images with quality-level labels are consistent with human perception, they reflect only HVS-aware quality factors; distortion and the joint effects of saliency and distortion are not considered. Moreover, large-scale expanded datasets are typically prepared to assist in specific IQA tasks. The more similar the extended pretraining dataset is to the original IQA dataset for the target task, the more effectively it can support the IQA task. In this case, a “similar” dataset is an expanded dataset that fully reflects the influences of the HVS-related perceptual factors (saliency and distortion) as embodied in the IQA task of interest. The current algorithms [15,20] that use this approach mainly follow the lead of RankIQA [20]: they generate a series of distorted image versions by adding different levels of distortion to each original parent image (with uniform distortion for each image region) and assign different numerical-valued labels to them to reflect the overall quality level. Consequently, the quality degradation of each distorted image depends only on the level of the distortion added to the whole image. As a result, HVS-aware quality factors are not well embedded into the expanded database. Using this type of extended dataset to pretrain the network will simply cause it to learn that a greater level of distortion leads to greater quality degradation; the network will be unable to discern that salient regions are more important than nonsalient regions and that different regions contribute differently to the overall image quality. Obviously, this type of expansion does not result in an ideal pretraining dataset for IQA.

In this paper, we introduce saliency into the large-scale expansion method, with the aim of constructing DNN-based BIQA models that will be effective in various cases where image quality is crucial. The objective is to be able to automatically estimate image quality to assist in handling low-quality images during the capture process. Moreover, by virtue of the introduction of saliency, our proposed model can achieve better prediction accuracy for large-aperture images (with clear foregrounds and blurred backgrounds), which are currently popular. We propose a new approach for incorporating saliency into BIQA that is perfectly compatible with the large-scale data expansion approach to ensure the full consideration of HVS-related factors in the mapping process. Specifically, we introduce saliency factors through regional distortion, thereby conveniently combining saliency and distortion factors during the expansion of each image to generate a series of distorted image versions. Then, we use the information entropy to rank these images based on their quality to complete the labeling process. By constructing a more efficient pretraining tool for DNN-based BIQA, we improve the prediction performance of the final model. We use our generated large-scale dataset to pretrain a DNN (VGG-16) and then use the original small IQA dataset to fine-tune the pretrained model. Extensive experimental results obtained by applying the final model to four IQA databases demonstrate that compared with existing BIQA models, our proposed BIQA method achieves state-of-the-art performance, and it is effective on both synthetic and authentic distorted images. Therefore, we conclude that a data expansion approach that fully reflects HVS-aware quality factors is beneficial for IQA. This study presents a novel method for incorporating saliency into IQA tasks, namely, representing it as regional distortion.

Our contributions can be summarized as follows: (1) We introduce saliency into the large-scale expansion method in a manner that fully reflects the influence of HVS-aware factors on image quality, representing a new means of considering saliency in IQA. With the incorporation of the saliency factor, the proposed data expansion method overcomes the main drawback of its predecessor algorithm, RankIQA [20], which enables the learning of only the quality decline caused by the overall distortion level. Our approach enables the construction of an efficient pretraining dataset for DNN-based BIQA tasks and results in improved prediction accuracy compared to previous BIQA methods. (2) We propose a new data expansion method that fully reflects HVS-aware factors by generating distorted images based on both distortion and saliency and assigning labels based on entropy. This method successfully embeds the joint influence of saliency and distortion into a large-scale expanded distorted image dataset.

The remainder of this paper is organized as follows. Section 2 describes the important factors that affect image quality and explores how those factors affect human judgments of image quality. Section 3 introduces the proposed expansion method and describes its use in IQA in detail. Section 4 reports the experimental results and presents corresponding discussions. Finally, Section 5 offers conclusions.

## 2. Exploration of Functional HVS Aspects for Image Quality

As stated above, the main requirement for the expanded dataset is that it should be as similar as possible to the original IQA dataset. Therefore, to identify some features of good BIQA model design, we analyzed the influence of the three functional aspects of the HVS on human visual IQA. To improve the reliability of the results, all images considered below were taken from the IQA dataset, which consists of distorted images and subjective quality score labels that are often used as criteria based on the human visual perception mechanism.

### 2.1. The Influence of Saliency on Image Quality

As previously discussed, saliency is an important factor that influences image quality because when people observe an image, they tend to focus on the regions that contain the most relevant information in the visual scene. Previous HVS evaluation experiments with eye trackers [11,12,13,21] showed that the visual importance of different local regions varies when humans are estimating the visual quality of a whole image.

**Figure 1:**
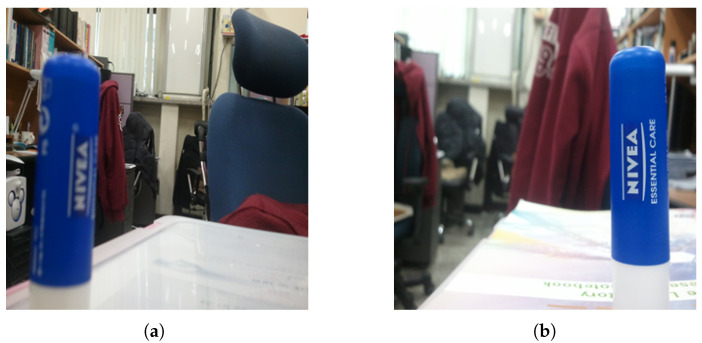
Distorted images from the LIVEC dataset: (**a**) MOS = 50.1882; (**b**) MOS = 72.4574.

To conduct a detailed analysis of the substantial impact of saliency on quality, we analyzed several images with different visual quality scores. The images shown in panels (a) and (b) of Figure 1 are derived from the LIVE Challenge dataset [22], an authentic distortion database in which the labels represent the mean opinion score (MOS) and take values in the range of [0, 100], with higher values indicating better quality; in this dataset, multiple nonuniform distortions typically appear in each image. Images (a) and (b) contain identical levels of blurring in the salient and nonsalient regions, respectively. However, image (b) has a much better visual quality label in the database than image (a) does. These examples show that the level of distortion in the salient regions of an image is more likely to determine the final quality rank than is the level of distortion in nonsalient regions. Humans can more easily perceive distortions in the salient regions and thus assign lower quality scores to images with such distortions. When the foreground area of an image is distorted, the visual quality score of the whole image immediately decreases, regardless of whether the background region is distorted. Thus, the quality in the salient regions is closely related to the final quality score of the whole image.

**Figure 2:**
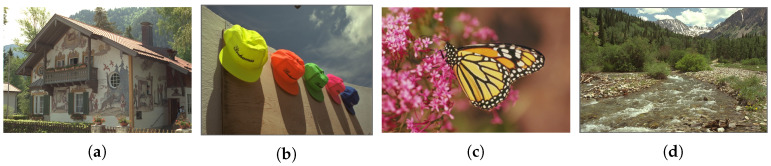
Reference images (DMOS = 0) from the LIVE database: (**a**) painthouse; (**b**) caps; (**c**) monarch; (**d**) stream.

By contrast, the effect in nonsalient regions is the opposite. As shown in Figure 1, the low level of distortion in the nonsalient regions of image (a) does not prevent the quality degradation caused by distortions in the salient region. This phenomenon is widespread, especially in synthetic distortion databases. The images shown in panels (a)–(d) of Figure 2 are from the LIVE [23] dataset, which is a synthetic distortion database that contains 29 reference images and 779 distorted images derived from them. The corresponding difference mean opinion score (DMOS) labels for these images, representing subjective quality scores, lie in the range of [0, 100], with a lower value indicating better visual quality. Images (a)–(d) contain no distortion in the salient regions and exhibit varying distortion intensities in the nonsalient regions. However, all of these images have the highest possible DMOS value of 0. This indicates that distortion in nonsalient regions attracts little attention and has little effect on the quality of the entire image.

### 2.2. The Influence of Content on Image Quality

**Figure 3:**
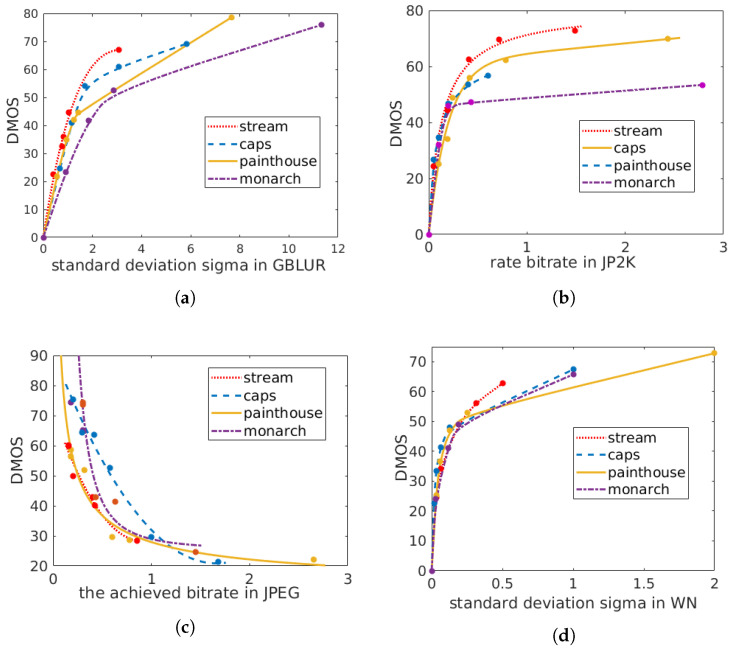
Relationship between the distortion parameter (x-axis) and the DMOS label (y-axis) for different distortion types. Each *x*-axis represents the distortion parameter for the corresponding distortion type. Each scatter point represents one sample in the LIVE dataset [23]. The scatter points representing the distorted images derived from the same reference image were separately fitted to a smooth curve. Different colors indicate different images: (**a**) GB; (**b**) JP2K; (**c**) JPEG; (**d**) WN.

We will now discuss the crucial impact of content on IQA. We present detailed figures for observation. Among the existing IQA databases, LIVE is the most commonly used. Its 29 reference images were distorted using five types of distortion: JPEG2000 (JP2K), JPEG, white noise in the RGB components (WN), Gaussian blur (GB), and transmission errors in the JPEG2000 bit stream using a fast-fading Rayleigh channel model (FF). Moreover, different levels of distortion were added to each reference image using the same distortion type to ensure that the quality of the distorted images of the same distortion type covers the entire quality range. To draw our conclusions, we selected 4 of the 29 reference images (“painthouse”, “caps”, “monarch” and “stream”, as shown in Figure 2) as well as the distorted images derived from these 4 reference images, as shown in Figure 3. Only 4 of the distortion types (JP2K, JPEG, WN, and GB), all of which are commonly used in IQA databases, are considered here. For each distortion type, we observed the distortion parameter and the DMOS label for each distorted image derived from the 4 reference images. We first generated a scatter plot showing the distortion parameters and the corresponding DMOS quality labels. Then, for each reference image, we artificially fit a smooth curve to these scatter points to observe the trend of variation relating the image quality and the perceived level of distortion.

First, we observe that there are no rating biases associated with the reference image contents; all of the reference images, each with different contents, are assigned the same quality score (DMOS = 0) in the public IQA database. This phenomenon is clearly reflected in Figure 3; the starting points of all curves in the same figure are consistent (because the x-axis of the figure for JPEG distortion represents the achieved bitrate, this characteristic is not reflected in this figure). Second, we find that as the level of distortion added to the image increases, images with different contents have different quality degradation curves. In other words, different image contents have different capacities for hiding distortion. For example, image (d) in Figure 2 has a dark content, and the slight distortion in it is unacceptable to the human eye. However, a further increase in the distortion level does not strongly affect an observer’s understanding of the content of “monarch”; therefore, the rate of quality degradation is slow.

### 2.3. The Influence of Distortion on Image Quality

The degree of distortion seriously affects the image quality. This conclusion is obvious and beyond doubt for all four distortion types displayed in Figure 3. For the same type of distortion, all images with different contents exhibit the same behavior: when the distortion added to the whole image is uniform, the higher the level of distortion (distinguishable by the human eye) added to the whole image is, the lower the image quality is. This means that a negative correlation exists between the level of distortion and the image quality. The same conclusion can be drawn from Figure 3.

### 2.4. Conclusion: The Joint Influence of Saliency and Distortion on Image Quality Given the Same Type of Distortion for Each Image

The above analysis provides the following inspiration. When a DNN learns a mapping between distorted images and quality scores, it is actually learning different curves for different image contents. This suggests that it should be possible to construct an expanded training database to improve the DNN-based prediction performance on BIQA tasks by simply adding synthetic distortions to a baseline database containing a large number of original images, exactly as was done to construct the LIVE database. As discussed above, the original external content is not subject to any rating biases; therefore, we should find a parent database that consists of multiple types of images with no distortion and then distort them using several distortion types. For each type of distortion, we could generate a series of distorted images of different qualities for each original image such that the generated distorted images would reflect the joint effects of saliency and distortion on image quality. Then, we could apply a reasonable two-stage training method. First, pairs of images of different qualities from the expanded dataset would be sent to the DNN to pretrain the network to learn the quality ranking of distorted images with the same content. Then, the smaller original IQA database could be used to fine-tune the DNN, which would already be trained to perform quality ranking, to refine the mapping of distorted images to quality scores for each type of content. Then, the DNN should be able to output high-precision score prediction results. The authors of the RankIQA algorithm [20] accomplished this task using four distortion types (JP2K, JPEG, WN, and GB) because these four types can be implemented by means of MATLAB functions and frequently appear in IQA datasets; they generated a series of distorted images from the contents of each original image separately. However, in their expanded dataset, the degradation of image quality with a given distortion type for each parent image depends only on the overall distortion level; the joint influence of distortion and saliency on image quality is not reflected. Thus, if we could fully capture the influence of both saliency and distortion in the expanded dataset, the performance should improve.

## 3. Proposed Method

**Figure 4:**
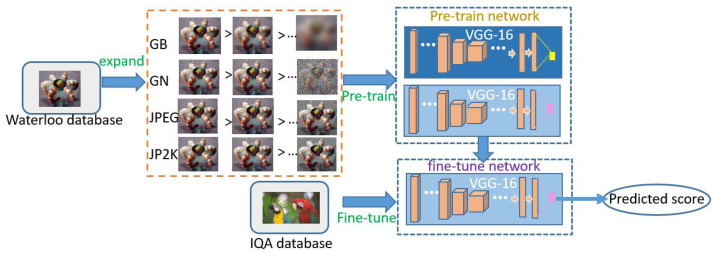
Pipeline of the proposed data expansion method for IQA. Based on the Waterloo database, we generate a large-scale expanded dataset, and this expanded dataset is then used to pretrain a double-branch network. Then, the original IQA dataset is used to fine-tune a single branch of the network to output quality scores.

As mentioned above, our main goal is to construct a newly expanded dataset to support DNN-based BIQA tasks. We introduce saliency into the large-scale expansion strategy for the first time by creating distorted images based on the joint consideration of both saliency and distortion. Finally, we label the images based on the information entropy. The degradation of image quality in our new expanded dataset not only is related to the distortion level (as in RankIQA [20]) but also fully reflects the joint influence of distortion and saliency on image quality. We use this large-scale expanded dataset to pretrain a DNN and then use the original small IQA dataset to fine-tune the pretrained DNN. After fine-tuning, we obtain the final BIQA model. The flow chart of our proposed method is shown in Figure 4.

In this section, we present a detailed description of our method, which is divided into two main stages: dataset expansion and the use of the expanded dataset. First, we introduce our novel method of incorporating saliency into the large-scale dataset expansion process for IQA. Then, we describe the dataset generation process: image expansion based on saliency and distortion and image labeling guided by the information entropy. Finally, we describe how the expanded dataset is used in the IQA task, which involves a two-step training process to ensure that the DNN fully learns how HVS-aware factors influence image quality.

### 3.1. The Usage of Saliency in IQA

**Figure 5:**
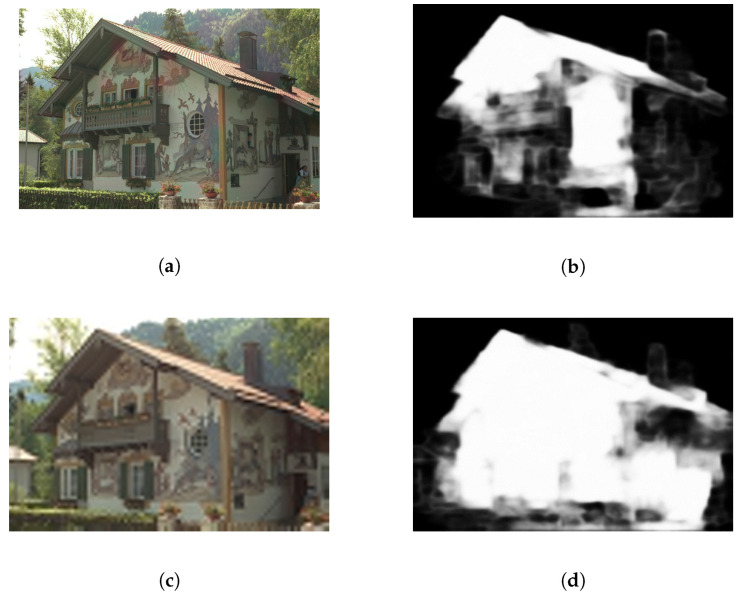
Saliency shift caused by different levels of blur. Two images with the same content but different distortion levels are shown on the left. The corresponding saliency maps are shown on the right. (**a**) “painthouse” with low level’s distortion; (**b**) the saliency map of (**a**); (**c**) “painthouse” with high level’s distortion; (**d**) the saliency map of (**c**).

The incorporation of saliency into the expansion procedure is a key step because we want to consciously capture the influences of both saliency and distortion when generating distorted images. Previous algorithms [16,17,18,19] introduced saliency into the IQA task by assigning different weights to different regions of a distorted image when predicting the final score. Such saliency usage is suitable for small-scale expansion but cannot be applied in the case of large-scale expansion. Moreover, there is no opportunity to add saliency factors to the existing distorted image versions generated for RankIQA (large-scale expansion), for which several images with different distortion intensities were created and labeled by quality rank. Because each label is a simple number that represents the overall quality level, using regional saliency weights is insufficient. Moreover, the salient regions in any given image may shift under different distortion levels; examples of this attentional shift based on distortion are shown in Figure 5. As the level of distortion increases, the salient areas also shift. Thus, we can see that differently distorted images with the same content should have different local saliency weight values. This saliency shift further increases the difficulty of adding saliency into the existing distorted images generated for RankIQA. Therefore, finding a new way to introduce saliency into the large-scale expansion process for IQA is crucial.

On the one hand, the characteristics of the large-scale expansion strategy are as follows: the time-consuming psychometric approach is not employed to obtain subjective score labels, and each distorted image derived from a given image by applying a given type of distortion has only a simple numerical label that represents its level of quality. On the other hand, Section 2.1 shows that the influences of salient and nonsalient regions on quality are quite different. Based on the two considerations above, we are inspired to introduce saliency into an expanded dataset in the form of regional distortion. We can generate multiple distorted images by adding distortion to high-resolution reference images. Among these distorted images, some will be subjected to global distortion of the original images, some will be distorted only in the salient regions of the reference images, and others will be distorted only in the nonsalient regions. Because the locality of the distortion (both regional and global) in the extended set of distorted images will be different, these images will have different perceptual qualities. Next, instead of asking volunteers to provide subjective scores, we can sort the distorted images based on their information entropy and assign simple numerical labels that represent their quality ranking. In this way, the combined effects of both saliency and distortion on quality will be reflected in the expanded dataset.

To implement the approach proposed above, we performed two preparatory steps. First, we needed to choose a saliency model. From among the many possible saliency models, we selected [24] because it emphasizes the identification of the entire salient area. Second, we needed to establish a measure of how the impact factor affects the quality (as discussed in Section 2). In addition to the information entropy, this will be another important measure for guiding the image generation and labeling processes during our expansion procedure. Based on these two preparatory steps, we introduce the details of our expansion method below.

### 3.2. Generating Images for the Expansion Dataset

**Figure 6:**
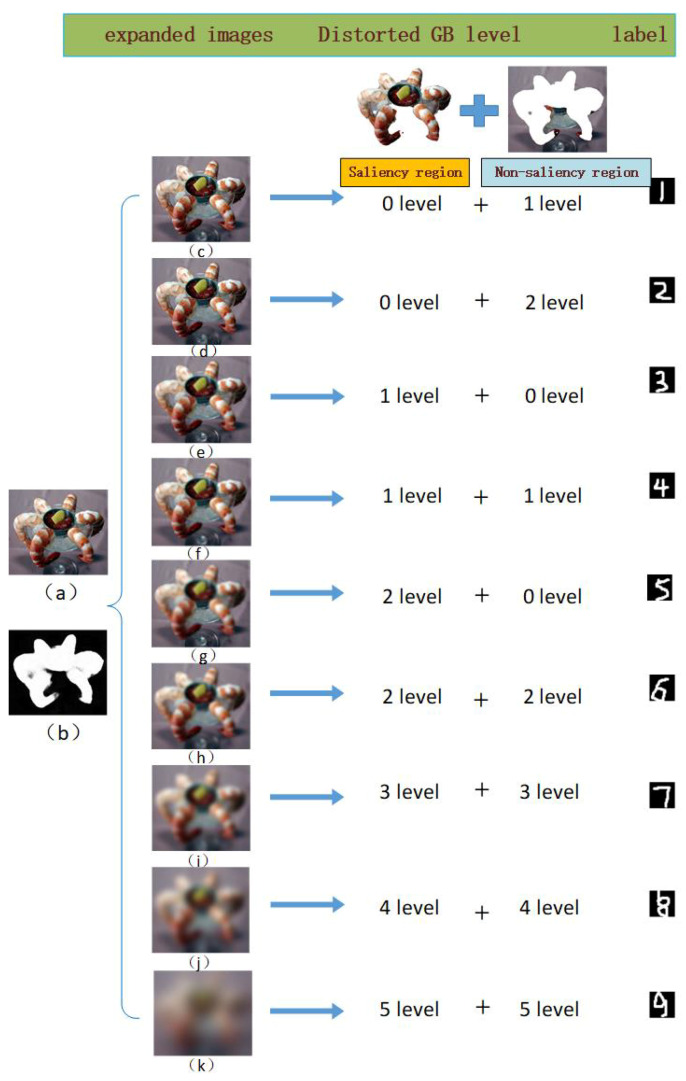
Image examples from our generated database. The first column contains an original image and its saliency map. The nine distorted images generated using GB distortion are displayed; each distortion was generated by adding particular levels of distortion to the salient and nonsalient regions of the original image. These distortion levels are displayed alongside the corresponding distorted images, and the corresponding image quality labels are given in the last column. Please note that the definitions of the salient regions and the distortion levels can be found in Section 3.2. Nine distorted images were generated in this way for all four distortion types, although only the results of GB expansion are shown in this figure. (**a**) the original version of “shrimp”; (**b**) the saliency map of (**a**); (**c**–**k**) the nine distorted versions of (**a**) under GB distortion type.

We selected the Waterloo database [25], which includes a large number of high-resolution images (4744), as the parent database to be used in the expansion process. Using MATLAB, we added distortion to these images to construct a large-scale expanded dataset containing a total of 4744 × 4 × 9 distorted images. Here, the factor of 4 arises from the 4 types of distortion (JP2K, JPEG, WN, and GB) applied to each parent image; we adopted these four distortion types because they are found in most available IQA databases. The factor of 9 arises from the fact that for each distortion type, a total of nine distorted images of different qualities were generated, using a total of five distortion levels for each distortion type. We summarize this information in Table 1. Please note that because we used MATLAB to simulate the types of distortion present in the LIVE dataset, the distortion functions and distortion factors used may be different from those used in LIVE; therefore, the parameters in Table 1 are slightly different from those in Figure 3. Next, with the help of Figure 6, we will explain how we used the five distortion levels and different saliency models to generate nine distorted versions of each parent image.

As an example, we chose one original parent image (“shrimp” from the Waterloo database), and the image shown in panel (b) is its saliency map, generated as described in [24]. Due to space constraints, only the nine distorted images generated using GB distortion are shown in Figure 6. Please note that nine corresponding distorted image versions were also generated for each of the other three distortion types from each original parent image. As Figure 6 shows, during the expansion procedure, we used the method introduced in [24] to extract the saliency map of each original parent image. Then, according to the saliency map, we defined the region with pixel values greater than 30 as the salient region and defined the remaining area as the nonsalient region. Each image was thus divided into two parts, the salient region and the nonsalient region. Then, we independently added different levels of distortion to these two regions of the original image and spliced the results to obtain a distorted image. The distortion levels applied to the salient and nonsalient regions to generate the nine distorted images are shown in the GB distortion level column (e.g., “level 0 + level 1” for image (c) means that this image was generated by adding GB distortion of level 0 to the salient region and GB distortion of level 1 to the nonsalient region of image (a)). The definitions of distortion levels 1–5 for each distortion type can be found in Table 1, and a level of 0 means no distortion.

Our expanded set of distorted images fully reflects the influence of HVS-aware quality factors. The nine distorted image versions generated from each parent image contain different levels of distortion across the entire image region, thus representing the influence of the overall distortion level on quality. In addition, some distorted images have different levels of distortion in the salient and nonsalient regions, thus representing the joint influence of saliency and distortion on quality. We ranked the nine distorted images of the same distortion type generated from each original image separately. The corresponding distorted image versions of decreasing quality can fully reflect the quality degradation caused by various HVS-aware factors.

### 3.3. Entropy-Based Image Quality Ranking of the Expanded Dataset

After generating the distorted images, we next assigned quality labels to them. We know that each image in an IQA database will have an assigned quality score generated through a time-consuming psychometric experiment, an option that is unavailable to us, and is, in fact, unnecessary. Labels that simply reflect the quality ranking are sufficient to create the needed effect (as discussed in detail in Section 3.4). We refer to the nine distorted images of the same distortion type generated from the same parent image as a group; thus, there are a total of 4744 × 4 groups in our expanded dataset. We sorted the nine distorted images in each group separately by quality using the information entropy defined on the basis of Shannon’s theorem because the information entropy is a measure that reflects the richness of the information contained in an image. The larger the information entropy of an image is, the richer its information and the better its quality. Moreover, the information entropy value is sensitive to image distortion and quality. Distortion in the salient region will lead to a significant reduction in the entropy value. Therefore, the information entropy is a suitable basis for our labeling procedure. The formula is as follows:

(1) $$H=-\sum_{i=0}^{255}p_{i}*logp_{i}$$

where *H* represents the information entropy of the image and $p_{i}$ represents the proportion of pixels with a grayscale value of *i* in the grayscale version of the image. The ordering of the information entropy values reflects the quality ranking of a group of images. We used this formula to calculate the information entropy of each of the nine distorted images in one group and ranked these nine images in order of their information entropy values. Accordingly, labels 1–9 were assigned to represent the image quality ranking. As mentioned above, there are a total of 4744 × 4 groups in our expanded dataset. We use letters *c* to *k* to denote the distorted image versions generated to compose each group (where *c* represents the distorted image generated by adding no distortion to the salient region and level 1 distortion to the nonsalient region of the original image). For each of these nine distorted image versions, we calculated the average entropy for the corresponding 4744 × 4 images, as shown in Table 2. The information entropy ranking results for most groups are consistent with the average order listed in Table 2. For each group, the labels for distorted images *c* to *k* range from 1 to 9, representing their sequentially decreasing quality. For example, for the nine images in Figure 6, their entropy sequentially decreases in the order in which they are displayed; the labels range from 1 to 9. Some groups also exist in which the information entropy order is different from the average order displayed in Table 2; in most such cases, the entropy values of images *d* (in which only the nonsalient region is distorted at level 2) and *e* (in which only the salient region is distorted at level 1) are reversed. However, we still sort image *e* below image *d* in quality to emphasize the importance of the salient region.

These information entropy results are consistent with the previous conclusions regarding how HVS factors affect image quality. Images with only background distortion have higher quality indices than those with foreground distortion and whole-region distortion because distortion in only nonsignificant regions leads to only weak quality degradation due to the smaller entropy of nonsalient regions. Consequently, images with only foreground distortion and with overall distortion at the same level are of similar quality. In addition, as we discussed in Section 2, the quality of the salient regions is highly consistent with that of the whole image. Please note that for a few landscape images in the Waterloo database, which have no obvious salient regions, we treated the entire image as the salient region to avoid negative effects. Although no convincing quality score labels could be extracted for these images, we were still able to use the expanded database for our BIQA task by adopting a Siamese network and a corresponding training method, as discussed in the next section.

### 3.4. Using the Expansion Dataset for the IQA Task

**Figure 7:**
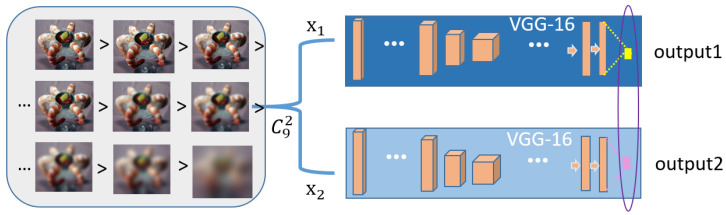
Pretraining process. The left side presents a series of distorted images of decreasing quality in the same group, and the right side presents a two-branch VGG-16 network where the two branches share parameters and a loss function.

Now, we will introduce the use of our new expanded dataset. Our training process consists of two steps: pretraining on the expanded dataset and fine-tuning on the IQA database. We trained a model based on VGG-16 [26], with the number of neurons in the output layer modified to 1. In our expanded database, for each original image, there are nine distorted images with corresponding labels from 1–9 that represent their quality ranking for each distortion type. We followed the training setup used by the authors of RankIQA [20]. During pretraining, to train the network on the quality ranking task, we used a double-branch version of VGG-16 (called a Siamese network) with shared parameters and a hinge loss. We show a schematic diagram of the pretraining process in Figure 7 and explain the training process in conjunction with the figure. Each input to the network consists of two images and two labels: a pair of images of different quality that are randomly selected from among the nine distorted images in one group. The image with the lower label (indicating higher quality) is always sent to the $x_{1}$ branch, and the other image is sent to the $x_{2}$ branch. When the outputs of the two branches are consistent with the order of the two labels, meaning that the network correctly ranks the two images by quality, the loss is 0. Otherwise, the loss is not 0, and the parameters will be adjusted (by decreasing the gradient of the higher branch and increasing the gradient of the lower branch) as follows:

(2) $$\frac{\partial L}{\partial \theta }=\left\{\begin{array}{cc} 0 & \mathrm{if}\;(f\left(x_{2};\theta \right)-f\left(x_{1};\theta \right))\leq 0,\\ \frac{\partial f(x_{2};\theta )}{\partial \theta }-\frac{\partial f(x_{1};\theta )}{\partial w} & \;\mathrm{otherwise}. \end{array}\right.\;$$

where θ represents the network parameters. Thus, the loss function is continuously optimized by comparing the outputs of the two branches, and eventually, the training of the quality ranking model is complete. Because any two of the nine distorted images in a group may be paired to form the input, the network is efficiently forced to learn the joint influence of saliency and distortion on image quality. After pretraining, either network branch can produce a value for an input image (because the two branches share parameters), and the quality ranking of different input images will be reflected by the order of their corresponding output values.

We have found that this pretrained model is nearly identical to the IQA model and can effectively judge the effects of saliency and distortion on quality. However, the output of this network is not a direct image quality score. Only when multiple different images are input to obtain different output values does the order of these values reflect the order of the images in terms of quality. Therefore, to facilitate the comparison of our model with other BIQA models and transform the network output into a direct quality score, our method includes an IQA-database-based fine-tuning step. From the pretrained model, we extract one branch to obtain a single VGG-16 network and perform training on the original IQA dataset to complete the fine-tuning process. In each round of training, the input to the network is one image, and the corresponding quality score is the label in the IQA database; thus, the network learns an accurate mapping from distorted images to scores. Again following the approach of RankIQA, we use the sum of the squared errors as the loss function during fine-tuning.

## 4. Experiments and Results

### 4.1. Datasets and Evaluation Protocols

We used two types of datasets in our experiments: a non-IQA dataset used for the generation of the large-scale expanded pretraining dataset and several IQA datasets for performing fine-tuning. As the non-IQA dataset that was used to generate new distorted images, we adopted the Waterloo Exploration Database [25], which includes 4744 high-resolution images. The diversity of the image scenes and the clarity of the images make this database suitable for our purposes. As the IQA datasets, we used three synthetic IQA databases (i.e., databases containing synthetic distortions), namely, LIVE [23], CSIQ [27], and LIVE MD [28], and one authentic IQA database, namely, LIVE Challenge (LIVEC) [22], in which the distortion present in each image may be a complex combination of multiple types (such as camera shaking and overexposure) to test our model’s generalization capability and its scope of application.

As the evaluation measures, we selected two metrics that are commonly used in the BIQA domain, namely, the Spearman rank order correlation coefficient (SROCC) and the Pearson linear correlation coefficient (PLCC). Given N input images, the SROCC is calculated as follows:

(3) $$SROCC=1-\frac{6\sum_{i=0}^{N}{\left(p_{i}-q_{i}\right)}^{2}}{N(N^{2}-1)}$$

first, the *N* ground-truth scores and *N* predicted scores are ranked separately. Accordingly, $p_{i}$ denotes the *i*-th value in the ordered list of predicted scores, and $q_{i}$ denotes the *i*-th value in the ordered list of ground-truth scores. Therefore, the SROCC measures the monotonicity of the predictions. The PLCC is calculated as follows:

(4) $$PLCC=\frac{\sum_{i=0}^{N}\left(u_{i}-\bar{u}\right)\left(v_{i}-\bar{v}\right)}{\sqrt{\sum_{i=0}^{N}{\left(u_{i}-\bar{u}\right)}^{2}}\sqrt{\sum_{i=0}^{N}{\left(v_{i}-\bar{v}\right)}^{2}}}$$

where $u_{i}$ and $v_{i}$ are the predicted score and ground-truth score, respectively, for the *i*-th image and *u* and *v* are the averages of the *N* predicted scores and the *N* ground-truth scores, respectively. Therefore, the PLCC measures the accuracy of the predictions. It can be seen from the formulas that the SROCC and PLCC both lie in the range of [0, 1] and that a larger value indicates a stronger correlation between the two columns of variables.

### 4.2. Experimental Setup

In Section 3.4, we introduced some information on the training process. Here, we provide more details and explain the reason for the selected experimental settings. To evaluate the performance improvement achieved by our algorithm in comparison with its predecessor algorithm RankIQA [20], we adopted the same network used in RankIQA—the VGG-16 architecture [26]—and changed the number of neurons in the output layer to 1 because our objective is not regression but rather a classification task. During both pretraining and fine-tuning, we randomly cropped a single subimage with dimensions of 224 × 224 from each training image to be used as the input in each epoch. During testing, we randomly sampled 30 224 × 224 subimages from one image and adopted the average of the corresponding 30 predicted outputs as the final score for this image. The quality ranking of the nine distorted images in a group was determined on the basis of an overall comparison of the full image region. Although a size of 224 × 224 is not sufficient to cover the entire image, it does cover more than 1/3 of the full image size; thus, this requirement does not destroy the quality ranking of the input images. Also for consistency with RankIQA [20], we adopted the Caffe [29] framework for training. The entire pretraining process consisted of 50,000 iterations, while the fine-tuning process consisted of 20,000 iterations. Additionally, L2 weight decay was used throughout the entire training process.

### 4.3. Performance Comparison

We compared the performance of our method on several IQA databases with the performance of various state-of-the-art FR-IQA and NR-IQA methods, including the FR-IQA methods PSNR, SSIM [1] and FSIMc [2]; the traditional NR-IQA methods BRISQUE [30], CORNIA [31], IL-NIQE [32] and FRISQUEE [33]; and the DNN-based NR-IQA methods CNN [10], RankIQA [20], BIECON [18], and DIQA [19]); as well as a DNN-based NR-IQA method that incorporates saliency, DIQaM [16]. We also compared our method with other well-known DNN models. Three networks (AlexNet [34], ResNet50 [35] and VGG-16, initialized from ImageNet) were also directly fine-tuned on each IQA database and treated as baselines. We used the final version of our DNN model, which was pretrained on the expanded dataset and then fine-tuned on the IQA dataset, to obtain image quality scores. The SROCC and PLCC were then calculated between the predicted quality scores (the output of our fine-tuned model) and the quality labels of the distorted images in the IQA database. The results are shown in Table 3, where the best three performance results are highlighted in bold. We divided the distorted images and their corresponding score labels into two groups, using 80% for training and 20% for testing. For all databases, the contents of the training and test sets did not overlap. This division process was repeated ten times. To avoid the influence of randomness on the evaluation of the prediction effect, the results of averaging the SROCC and PLCC scores over all ten runs are reported in Table 3.

First, it can be clearly seen that our proposed model achieves the highest PLCC and SROCC scores on almost all tested databases, indicating that the proposed data expansion method for DNN-based BIQA has the best overall effect for both synthetic and authentic distortion databases and is largely consistent with the subjective judgments made by humans. Moreover, we can see that compared with its predecessor algorithm, RankIQA, our method achieves better results on all of the datasets listed, especially on LIVEC, because the introduction of the saliency factor causes the model to somewhat depend on the distortion type consistency between the expanded dataset and the original IQA database. The performance improvement on CSIQ is also considerable, possibly because the reference images in this dataset include many examples with clear foregrounds but blurred backgrounds.

Table 3 also concisely presents a comparison of the different types of methods that can be applied to IQA tasks. We can see that our method is superior to any of the classical NR methods due to the strong autonomous learning capability of CNNs. Among the deep learning methods, many models performed poorly on the LIVEC dataset because their training process requires reference images, which do not exist in the LIVEC dataset. By contrast, our fine-tuning process does not require reference images. Moreover, from the results of the directly fine-tuned baselines listed above, we can see not only that a good algorithm can perform well but also that the convolutional computing ability of a relatively deep and large network such as ResNet50, which has a total of 50 layers, is advantageous. However, our approach of introducing an expanded dataset makes it easy to use a smaller network, which incurs lower computational costs, to achieve results similar to those of a larger network.

Because the SROCC and PLCC results were averaged over ten runs in our experiment, we also present the standard deviations of these results to illustrate the stability of our model’s predictive performance. Table 4 shows the standard deviations of the PLCC and SROCC scores over ten runs for RankIQA and our method. Because our method uses the same training procedure as RankIQA but differs in the use of the expanded dataset, RankIQA is a suitable choice for comparison. The other BIQA methods (whose specific experimental data are unavailable and which are also less suitable as methods for comparison) are not shown in Table 4. As Table 4 shows, the standard deviations of the results of our algorithm are smaller than those of RankIQA. This finding indicates that our algorithm achieves not only better prediction performance but also higher stability. Moreover, it is interesting to find that the performance on the LIVE MD dataset sometimes fluctuates across different divisions of the training and test datasets. These fluctuations may occur because some of the images contained in this dataset have unclear foregrounds, and when these images appear in the training set, they may induce a reduction in performance. Nevertheless, the average result is high. Therefore, the standard deviations of the prediction results further reflect the effectiveness of our proposed data expansion method.

### 4.4. Scatter Plots

**Figure 8:**
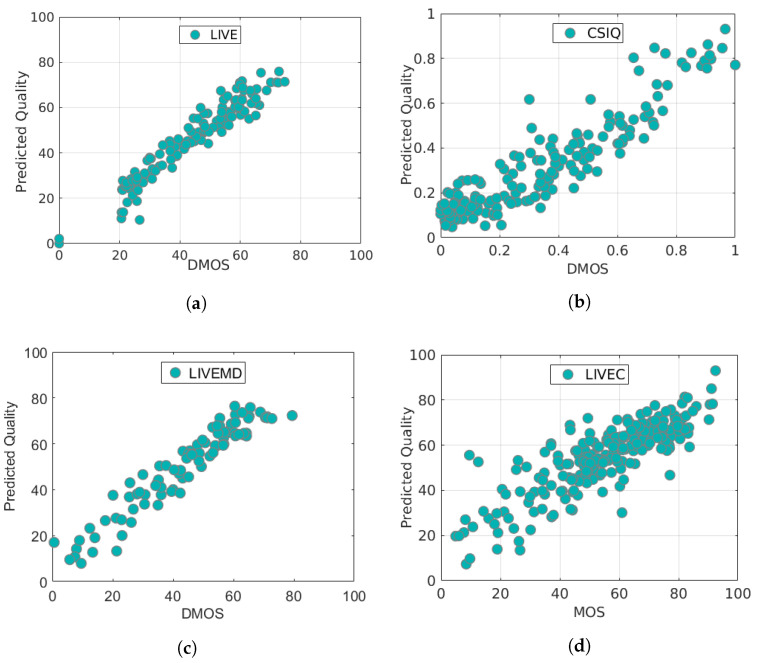
Scatter plots of the quality scores predicted by our method versus the ground-truth subjective DMOSs/MOSs for four datasets: (**a**) LIVE; (**b**) CSIQ; (**c**) LIVE MD; (**d**) LIVEC.

To further visualize the consistency between our method’s final predicted scores and the subjective human perception scores in the IQA databases, we show scatter plots of the scores predicted by our model (pretrained on the expanded dataset and fine-tuned on the corresponding IQA database) versus the ground-truth labels (DMOSs/MOSs) in Figure 8. This is another way of expressing the information in Table 3 and clearly shows the agreement between the predicted scores and the ground-truth values. Scatter plots of the results obtained on each of the four IQA databases (LIVE, CSIQ, LIVE MD and LIVEC) are shown in Figure 8. In these scatter plots, each point represents an image sample, the x-axis represents the DMOS/MOS scores associated with the samples in the dataset, and the *y*-axis represents the predicted quality scores obtained with our method. Because the four databases use different subjective score labels (i.e., LIVE and LIVE MD use DMOS scores in the range of [0, 100], LIVEC uses MOS scores in the range of [0, 100], and CSIQ uses DMOS scores in the range of [0, 1]), there are two different *x*-axis ranges in Figure 8. For the CSIQ database, the *x*- and *y*-axis scales range from 0 to 1. For the other three databases, unified scales from 0 to 100 are used.

Figure 8 shows that the predicted quality scores output by our method have a monotonic relationship with the ground-truth labels, especially on the LIVE MD and CSIQ datasets. This plot also explains the high correlation coefficients achieved on these two datasets. For the LIVEC dataset, the sample points are not tightly clustered around the isopleth, and the correlation is more obvious when the MOS value is small, which is unexpected due to the multiple distortion types and diversity of scenes. Nevertheless, the sample points for LIVEC are roughly evenly distributed on both sides of the isopleth, which represents great progress compared with the other algorithms. Thus, we can conclude that our expanded dataset provides effective support for IQA and gives the final model the capability to precisely predict human-perceived image quality over a wide range of datasets.

### 4.5. Ablation Studies

**Figure 9:**
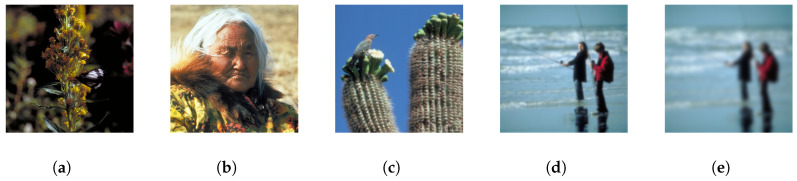
Distorted images from the CSIQ dataset. Their DMOS values increase from (**a**–**e**), representing a decrease in image quality.

The output of our pretrained model is not a direct image quality score. Only when multiple different images are input and their output values are obtained can the order of these values reflect the quality ranking of the images. To further evaluate the contribution of our expanded dataset and more accurately evaluate the contribution of the incorporation of saliency during the pretraining stage, we applied our pretrained model to various images and compared its predicted outputs to evaluate whether it could precisely rank images by quality. We compared our model with the pretrained RankIQA model, for which only images with whole-region distortion are considered during pretraining. Five image examples from the CSIQ database are shown in Figure 9, and in Table 5, we show the ground-truth label ranking, the order of the output of the pretrained RankIQA model, and the order of the output of our pretrained model for these images. We can see that RankIQA can accurately sort images (d) and (e), which contain whole-region distortion, but fails on images (a) and (c), which have clear foregrounds but blurred backgrounds accounting for nearly half of the entire image. By contrast, after only pretraining on the expanded dataset, our model fully reflects the joint influence of saliency and distortion and thus can perform well on images with both whole-region distortion and only local-region distortion, as is particularly evident from its performance on (a) and (c). We can see that the distortion level in the foreground in (c) is larger than that in (a), but the overall distortion of (c) is less than that of (a) when the entire image is considered. RankIQA [20] will tend to output a better quality score for (c) because the RankIQA model has only “distortion-level” awareness during training; it considers all regions equally in the final prediction. However, because our pretrained model has saliency awareness, it can sort the images correctly, as expected; therefore, our expanded database, which is based on both saliency and distortion and guided by information entropy, is more “similar” to the IQA database and can thus provide more effective assistance for the IQA task.

### 4.6. Discussion

#### 4.6.1. Studies on the Generation of Expanded Datasets from Different Parent Databases

In this section, we study the effects of using different parent databases for expansion. To confirm the effectiveness of our selected parent database, we performed tests using different databases as parents for data expansion, including the Waterloo database, which consists of images with rich scene contents, and MSRA-B [36], another classical database that contains 5000 original high-quality images and their saliency maps. The results can be seen in Table 6, where better performance results are highlighted in bold type. When we used MSRA-B as the baseline database to generate a series of distorted images for pretraining, the performance was reduced to a certain degree. This result was unexpected; however, it can be attributed to the insufficient richness of the MSRA-B dataset, which contains only images that are somewhat monotonous and have clear salient regions. By contrast, the complexity of the image content in the IQA datasets varies widely. Therefore, the more content-abundant Waterloo dataset was better suited to our requirements and resulted in higher performance.

#### 4.6.2. Studies on Generating Different Numbers of Distorted Images for Each Distortion Type

As mentioned in Section 3, we refer to the nine distorted images of the same distortion type that are generated from the same original image as a group. Here, we study the influence of generating different numbers of distorted images per group. We tested three different designs for the distorted images generated in the expansion process. The number “7” refers to a design in which we removed the second (no distortion added to the salient region and level 2 distortion added to the nonsalient region of the original image) and fifth (level 2 distortion added to the salient region and no distortion added to the nonsalient region of the original image) distorted images in each group, resulting in only seven distorted images per group. This approach results in the generation of a total of 4744 × 4 × 7 distorted images. The number “9” refers to the group design represented in Figure 6. This approach results in the generation of a total of 4744 × 4 × 9 distorted images. The number “11” refers to a design in which we added two further distorted images to each group—a distorted image obtained by adding no distortion to the salient region and level 3 distortion to the nonsalient region of the original image—and another distorted image obtained by adding level 3 distortion to the salient region and no distortion to the nonsalient region. These additional images were inserted after the second image and after the sixth image, respectively, of the previously described 9-image group. The results are shown in Table 7, where we highlight the best performance results in bold type. We can see that when these different expanded databases are used for pretraining, as the number of distorted images per group increases, the performance initially increases and then decreases. The highest value is reached in case “9”, possibly because of overfitting induced by the larger database, leading to reduced performance. When the number of distorted images per group increases past a certain threshold, the saliency effect becomes invalid and may lead to incorrect sorting. These findings indicate that the training process reaches saturation with the addition of two pairs of local-region distortions. Therefore, we elected to use nine distorted images per group, as shown in Figure 6.

## 5. Conclusions

In this paper, we have proposed a new approach for considering saliency in IQA. In this approach, we expand a large-scale distorted image dataset with HVS-aware labels to assist in training a DNN model to more effectively address IQA tasks. The novel feature of the proposed method is that this is the first time that a saliency factor was incorporated into the large-scale expansion strategy by representing saliency the form of a regional distortion. Then, by using the information entropy to rank the generated images by quality, we ensure that the labels in the newly expanded dataset are highly consistent with human perception. The ability to fully consider the various factors affecting image quality also solves the overfitting problem. Specifically, the introduction of saliency not only improves the applicability and versatility of the overall model but also overcomes the heavy reliance of the predecessor to our algorithm on the degree of similarity between the distortion types in the expanded dataset and the original IQA database. The final experimental results demonstrate the effectiveness of the proposed method, which outperforms other advanced BIQA methods on several IQA databases.

## Figures and Tables

**Table 1:** Important indicators and parameters involved in the expansion process. Distortion levels 1–5 are defined based on the distortion parameters used in the expansion procedure. The five distortion parameters presented for each distortion type are listed in order from level 1 to level 5.

Parent Database	Distortion Type	Distortion Level of 1–5 Involved	Expanded Numbers
Waterloo [25]	GB	Gaussian filter factor: 7, 15, 39, 91, 199.	9
	WN	Gaussian white noise: the mean is 0, and the variance is	9
		$2^{-10}$, $2^{-7.5}$, $2^{-5.5}$, $2^{-3.5}$, $2^{0}$.	
	JP2K	Quality factor: normalized variance at 43, 12, 7, 4, 0.	9
	JPEG	compression ratio factors: 0.46, 0.16, 0.07, 0.04, 0.02	9

**Table 2:** The average information entropy values for all images corresponding to the same distorted image version across all groups. Each value is calculated as the average information entropy of the corresponding image version in each of the 4744 × 4 groups.

Distorted Version	c	d	e	f	g	h	i	j	k
H(entropy)	7.5129	7.5087	7.5079	7.5074	7.4920	7.4850	7.3964	7.1882	6.8059

**Table 3:** Comparison of the SROCC and PLCC scores on the four IQA datasets.

Types	Algorithms	LIVE		CSIQ		LIVE MD		LIVEC	
		SROCC	PLCC	SROCC	PLCC	SROCC	PLCC	SROCC	PLCC
FR	PSNR	0.876	0.872	0.806	0.800	0.725	0.815	N/A	N/A
	SSIM	0.913	0.945	0.834	0.861	0.845	0.882	N/A	N/A
	FSIMc	0.963	0.960	0.913	0.919	0.863	0.818	N/A	N/A
NR	CORNIA	0.942	0.943	0.714	0.781	0.900	0.915	0.618	0.662
	BRISQUE	0.939	0.942	0.775	0.817	0.897	0.921	0.607	0.645
	IL-NIQE	0.902	0.908	0.821	0.865	0.902	0.914	0.594	0.589
	FRIQUEE	0.948	0.962	0.669	0.704	0.925	0.940	0.720	0.720
	*AlexNet*	0.942	0.933	0.647	0.681	0.881	0.899	**0.765**	**0.788**
	*VGG-16*	0.952	0.949	0.762	0.814	0.884	0.900	0.753	0.794
	*ResNet50*	0.950	0.954	**0.876**	**0.905**	0.909	0.920	**0.809**	**0.826**
	*CNN*	0.956	0.953	0.683	0.754	**0.933**	0.927	0.516	0.536
	*RANK*	**0.981**	**0.982**	0.861	0.893	0.908	0.929	0.641	0.675
	*BIECON*	0.961	0.960	0.815	0.823	0.909	**0.933**	0.663	0.705
	*DIQaM*	0.960	**0.972**	**0.869**	**0.894**	0.906	**0.931**	0.606	0.601
	*DIQA*	**0.970**	**0.972**	0.844	0.880	**0.920**	**0.933**	0.703	0.704
	*ours*	**0.978**	**0.983**	**0.893**	**0.916**	**0.935**	**0.947**	**0.818**	**0.837**

Red: the highest. Blue: the second. Green: the third.

**Table 4:** Standard deviations of the SROCC and PLCC scores for RankIQA and our method.

Standard Deviation	RankIQA		Our Method	
	SROCC	PLCC	SROCC	PLCC
LIVE	0.0106	0.0152	0.0057	0.0095
CSIQ	0.0131	0.0119	0.0125	0.0093
LIVE MD	0.0584	0.0454	0.0482	0.0303
LIVEC	0.0340	0.0309	0.0073	0.0106

**Table 5:** The ranking orders for several images as obtained with the pretrained models of RankIQA and our method. A larger value represents a worse image quality.

Algorithms	(a)	(b)	(c)	(d)	(e)
label ranking	1	2	3	4	5
ranking of RankIQA [20]	3	1	2	4	5
ranking of ours	1	2	3	4	5

**Table 6:** Comparison of the SROCC and PLCC scores of fine-tuned models pretrained on different expanded datasets.

Database	LIVE		CSIQ		LIVE MD		LIVEC	
	SROCC	PLCC	SROCC	PLCC	SROCC	PLCC	SROCC	PLCC
Waterloo	**0.978**	**0.983**	**0.893**	**0.916**	**0.935**	**0.947**	**0.818**	0.837
MSRA-B	0.975	0.972	0.871	0.896	0.907	0.923	**0.818**	**0.841**

**Table 7:** Performance differences caused by generating different numbers of distorted images per parent image for each distortion type.

Number	LIVE		CSIQ		LIVE MD		LIVEC	
	SROCC	PLCC	SROCC	PLCC	SROCC	PLCC	SROCC	PLCC
7	0.974	0.971	0.874	0.908	0.931	0.944	**0.825**	0.795
9	**0.978**	**0.983**	**0.893**	**0.916**	**0.935**	**0.947**	0.818	**0.837**
11	0.975	0.976	0.868	0.874	0.935	0.923	0.808	0.807
